# Inadequate nutrient intakes in Filipino schoolchildren and adolescents are common among those from rural areas and poor families

**DOI:** 10.29219/fnr.v63.3435

**Published:** 2019-05-16

**Authors:** Imelda Angeles-Agdeppa, Liya Denney, Marvin B. Toledo, Virgilyn Anne Obligar, Emma F. Jacquier, Alicia L. Carriquiry, Mario V. Capanzana

**Affiliations:** 1 Food and Nutrition Research Institute, Department of Science and Technology, Taguig City 1631, The Philippines; 2 Nestlé Research, Vers-chez-les-Blanc, Lausanne 1000, Switzerland Nestlé Research Centre, Lausanne, Switzerland; 3 Iowa State University, Ames, IA, USA

**Keywords:** usual nutrient intakes, school-aged children, adolescents, the Philippines

## Abstract

**Background:**

Adequate nutrition during childhood and adolescence is essential to promote growth and development.

**Objective:**

This study evaluated usual energy and nutrient intakes of Filipino schoolchildren and adolescents.

**Design:**

Food and beverage intakes were collected from a nationally representative sample of schoolchildren aged 6–9 and 10–12 years (*n* = 3,594 and *n* = 2,971, respectively) and adolescents aged 13–18 years (*n* = 5,447) using 24-h dietary recalls. The distributions of usual energy and nutrient intakes and the prevalence of inadequate intakes, which is defined as the percent of children with intakes less than estimated average requirements or acceptable macronutrient distribution ranges, were estimated using the Personal Computer Software for Intake Distribution Estimation (PC-SIDE) program.

**Results:**

The results showed that the mean energy intakes were 19–35% lower than estimated energy requirement. High prevalence of inadequate intakes was found for most macro- and micronutrients. Prevalence of inadequacies was as follows: protein 12–47%, total fat (as percentage of energy) 38–52%, calcium 92–94%, iron 75–90%, vitamin C 68–96%, folate 61–93%, vitamin A 58–81%, riboflavin 58–91%, thiamin 27–75%, and phosphorus 18–91%.

**Conclusions:**

Generally, prevalence of inadequacy of key nutrients were relatively high among adolescents and schoolchildren, especially those from poor families and rural areas. This study demonstrated that nutrient intakes of Filipino schoolchildren and adolescents were highly inadequate, particularly among the poor and those living in rural areas.

## Popular scientific summary

- Adequate nutrition during childhood and adolescence is essential to promote growth and development.
- This study evaluated usual energy and nutrient intakes of Filipino schoolchildren and adolescents.
- The results demonstrated that intakes of many key nutrients in this population were highly inadequate, particularly among the poor and those living in rural areas.
- The findings could be used by government agencies, non-profit organizations, and policy makers to develop intervention programs addressing the nutritional needs of this population.

Childhood and adolescence are both stages of physical, social, and cognitive growth and development (1, 2). Nutrient needs tend to parallel with rates of growth. Growth continues at a steady rate during childhood and then accelerates during adolescence; hence, there is a need for increase in nutrient intake. It is vital that children are provided with a diet containing sufficient quantities of macro- and micronutrients to allow them reach their growth and development potential (3). Inadequate intake of energy, protein, or certain micronutrients is reflected in slow growth rates, cognitive deficits, poorer school performance, and inadequate bone mass (4). However, little is known about the nutrition of adolescents, particularly in low-and middle-income countries. Dietary intake data for schoolchildren and adolescents are critical to guide appropriate intervention programs to improve their health and growth.

A number of studies have demonstrated that socioeconomic status (SES), usually measured as income, education, and occupation, contributes to inequalities in health and nutrition across all age groups (5). On average, a higher socioeconomic level has been associated with healthier dietary patterns in the developed countries (6), while persons of low SES are at risk of food insecurity and malnutrition (7, 8). Although most of these studies were focused on adults, however, low SES has been found to be associated with poor dietary patterns in children and adolescents (6). Apart from SES, place of residence may also affect nutritional status, as shown by the gaps in nutrient intakes observed between urban and rural areas (9). This is likely due to the accessibility and availability of a wide variety of food options in urban areas compared with rural areas (10).

In the Philippines, children and adolescents constitute almost one-third of the population (11). However, data from the latest Philippine National Nutrition Survey conducted in 2013 (2013 NNS) revealed that among children aged 5–10 years, 3 out of 10 were underweight and stunted, and among adolescents aged 10–19 years, 3 out of 10 were also stunted, and 12.4% were wasted. The highest prevalence of stunting, underweight, or wasting was found among those from poor families and rural areas (12). On the other hand, among the children aged 5–10 years from rich families, 1 in 4 was overweight or obese, and among 10–19-year-olds, 8.3% were overweight or obese, showing a 70% increase compared with the 4.9% in 2003. Furthermore, studies on worldwide trends in food consumption, including the Philippines, indicated that food consumption habits of Filipino children and adolescents have changed over the past few decades. These children and adolescents now consume more fat, especially saturated fats, and sweetened beverages and at the same time are not eating enough fruits or vegetables, thereby resulting in inadequate fiber consumption and inadequate intakes of other nutrients (13, 14).

Currently, a comprehensive assessment of usual intakes of energy and nutrients in a national representative sample of Filipino schoolchildren and adolescents is lacking. This knowledge can help understand the causes of malnutrition in this population and provide a direction to develop intervention strategies. The aims of this study were to evaluate the usual energy and nutrient intakes of Filipino schoolchildren and adolescents and also to look into how some socioeconomic factors, including place of residence and wealth status of families, affect their intakes.

## Material and methods

### Study population

The 2013 NNS is a cross-sectional and population-based survey conducted nationwide, covering all the 17 regions and 80 provinces in the Philippines. The survey used a multi-staged stratified sampling system. A total of 8,592 households were sampled for dietary survey, with a response rate of 87.7%. Individual dietary intake data from 12,012 schoolchildren aged 6–12 years (*n* = 6,565) and adolescents aged 13–18 years (*n* = 5,447) from the households were used in the current study. The Ethics Committee of the Food and Nutrition Research Institute (FNRI) approved the survey protocol. All surveyed households provided informed consent prior to participation.

### Data collection

To estimate the day-to-day variation in energy and nutrient intakes, two 24-h dietary recalls were conducted by registered dietitians with the parents and children face-to-face using structured questionnaires. The first 24-h dietary recall was collected for all sampled households, and a second 24-h dietary recall was repeated in 50% of randomly selected households on a non-consecutive day. The second 24-h dietary recall was typically collected 2 days after the first 24-h recall. All food and beverages that the child consumed on the previous day were recorded during dietary recalls. The amount of each food item or beverage was estimated using common household measurements, such as cups, tablespoons, by size, or number of pieces. The information was then converted to grams using a portion to weight list for common foods compiled by FNRI or through actual weighing of food samples.

Socioeconomic and demographic data and anthropometric parameters of growth were taken from the 2013 NNS. The wealth status of families was classified by wealth quintiles, a composite measure of a household’s ownership of selected assets including televisions, bicycles, materials used for housing construction, and types of water access and sanitation facilities. Scores were generated for each household asset and were then used to define wealth quintiles as poorest, poor, middle, rich, and richest. Weight and height were measured using mechanical Detecto^®^ platform beam balance scales (Detecto, Webb City, Missouri, USA) and Microtoise (SECA 206, Hamburg, Germany), respectively. Nutritional status indices such as stunting, wasting, and overweight were determined using the World Health Organization Child Growth Standards (15).

### Data processing

A computer system called Individual Dietary Evaluation System developed by FNRI was used to evaluate the energy and nutrient content of foods consumed by each individual subject. This system contains the data of the expanded Filipino Food Composition Tables (FCT) created for this study (16). The expended FCT contains 27 nutrients from 1,359 foods.

Food coding and quantity recorded were reviewed to avoid misclassification and under- or overestimation. Energy and nutrients intakes obtained were also scanned to identify implausible values. Estimated energy requirements (EER) were calculated using the equations from the Institute of Medicine considering age, sex, body weight, height, and physical activity level (17). We assumed low physical activity for schoolchildren aged 6–9 years and active physical activity level for schoolchildren aged 10–12 years and adolescents aged 13–18 years. For the evaluation of energy intake, the ratio of daily energy intake to EER was calculated for each individual and transformed into a logarithmic scale to remove outliers below −3 standard deviations (SDs) and above +3 SDs (18). After the checking, 33 individuals were excluded from the analysis for energy intake. For the evaluation of micronutrient intakes, excessive micronutrient intakes were defined as those that exceed 1.5 times the 99th percentile of the observed intake distribution in the corresponding sex and age group. Intakes above this upper limit were substituted by a random value generated from a uniform distribution in the interval with lower bound equal to the 95th percentile of observed intake and an upper bound equal to 1.5 times the 99th percentile (18).

### Statistical analysis

Participants of both sexes were grouped into three age groups: schoolchildren aged 6–9 years, schoolchildren aged 10–12 years, and adolescents aged 13–18 years. Mean and percentiles of usual intake distributions of nutrients were estimated by using the PC-SIDE software version 1.0 developed by Iowa State University (ISU) (Iowa State University, Ames, IA, USA). The ISU method adjusts daily intakes to remove the effect of intraindividual variability; therefore, the estimated distribution only reflects between-person variability in intake (19). This program also estimates the proportion below estimated average requirements (EARs) (20). In this study, The EARs used were from the Philippine Dietary Reference Intakes 2015 (21).

Acceptable macronutrient distribution ranges (AMDRs) were used to evaluate carbohydrates, total fat, and protein intakes as a percentage of energy. The proportions of inadequate and excessive intakes were estimated as less than AMDR lower range and greater than AMDR upper range, respectively. Tolerable upper intake level (UL) was used to estimate the proportions of excessive nutrient intakes. For the inadequate intake of iron, a probability approach was used (22). Individual iron intakes were rescaled assuming 8% bioavailability (21), and then the risk of inadequacy was computed. The prevalence of inadequate iron intake was the average risk of inadequacy in the group.

Calculations for summary statistics and differences between groups were carried out using Stata (StataCorp 2017 Stata Statistical Software, release 15, College Station, TX, USA). T-tests were performed to examine the differences between rural and urban groups. Differences between wealth quintiles were analyzed using Analysis of variance (ANOVA) and Bonferroni multiple comparisons. All analyses accounted for the complex survey design and sampling to reflect nationally representative results.

## Results

### Demographic characteristics of the study population

Table 1 presents the characteristics of the study population. Of the total participants, 52% were boys, and the rest were girls. More than half of the schoolchildren and adolescents lived in urban areas (58%), while the rest lived in rural areas. Approximately, 51% of the respondents belonged to the poorest (28%) and poor (23%) wealth quintiles. Most mothers were unemployed (58%) and had an education of high school or vocational level. Among the study participants, the prevalence of stunting and wasting was 16.6 and 6.5% in schoolchildren aged 6–12 years and 13.7 and 5.2% in adolescents, respectively. In addition, overweight was 2.8% among schoolchildren aged 6–12 years and 2.3% among adolescents.

**Table 1:** Characteristics of the study population

Respondents characteristics	Description	*n*	%
Sex	Boys	6,264	52.1
	Girls	5,748	47.9
Age	6–9 years old (schoolchildren)	3,594	29.9
	10–12 years old (preteens)	2,971	24.7
	13–18 years old (teenagers)	5,447	45.3
Wealth quintiles	Poorest	3,238	27.7
	Poor	2,690	23.0
	Middle	2,250	19.2
	Rich	1,883	16.1
	Richest	1,629	13.9
Urbanity	Urban	6,934	57.7
	Rural	5,078	42.2
Stunting	6–9 years old	1,018	8.6
	10–12 years old	937	8.0
	13–18 years old	1,607	13.7
	All	3,562	30.3
Wasting	6–9 years old	339	2.9
	10–12 years old	424	3.6
	13–15 years old	607	5.2
	All	1,370	11.6
Overweight	6–9 years old	158	1.3
	10–12 years old	172	1.5
	13–15 years old	267	2.3
	All	597	5.1
Body weight (kg)	6–9 years old	21.57	0.09
	10–12 years old	31.3	0.16
	13–18 years old	45.6	0.13
	All	34.8	0.12
Height (cm)	6–9 years old	119.2	0.14
	10–12 years old	137.2	0.17
	13–18 years old	154.5	0.12
	All	139.6	0.16
Mother’s education	No grades completed	170	2
	Elementary level	1,350	16.1
	High school level	2,993	35.7
	Vocational level	2,240	26.7
	College level	856	10.2
	Others (Special education (SPED), Arabic schooling, etc.)	778	9.3
Mother’s current work	No occupation	4,803	58.6
	With job/business	3,389	41.4

### Intakes of energy and nutrients

Inadequate intakes of energy and macronutrients were found in all age groups, and the extent of inadequacies increased with age. In the order of schoolchildren aged 6–9 years, schoolchildren aged 10–12 years, and adolescents, the mean energy intakes of these groups were 19, 29, and 35% lower than the estimated EERs, respectively. The prevalence of inadequate total fat intake, which is evaluated as the percentage of energy below the AMDR, was 38, 49, and 52%, respectively. The prevalence of inadequate protein intake was 12, 21, and 47%, respectively (Tables 2–4).

**Table 2:** Usual energy and nutrient intakes from food and beverages for Filipino schoolchildren aged 6−9 years from 2013 NNS (*n* = 3,594)

Nutrients	Dietary reference intakes^1^				Mean/median intake percentiles					Inadequate/excessive reported intake	
	EAR/AMDR	AI/RNI	UL	10th	25th	Median	Mean ± SE	75th	90th	% < EAR/AMDR	%>AMDR/>UL
**Macronutrients**											
Energy intake (kcal/d)	1,474 (EER)			762	941	1,184	1242.6 ± 7	1,479	1,798	-	-
Total fat (g/d)	-	-	-	9.7	15	23.1	26.1 ± 0.3	33.7	46	-	-
Saturated fat (g/d)	-	-	-	4	6	10	12.8 ± 0.2	15	24	-	-
Protein (g/d)	24	-	-	23.2	29	36.8	38.8 ± 0.2	46.3	56.7	12	-
Carbohydrate (g/d)	-	-	-	131	162	202	213.8 ± 1.3	253	310	-	-
Total sugar (g/d)	-	-	-	9.3	15.1	23.6	27.1 ± 0.3	34.8	48.6	-	-
Dietary fiber (g/d)	-	11–14	-	3.7	4.6	5.8	6.3 ± 0.04	7.5	9.4	-	-
**As percentage of total energy**											
Total fat (%)	15–30^a^	-	-	9.1	12.6	17.2	17.8 ± 0.1	22.3	27.2	38	5
Protein (%)	6–15^a^	-	-	10.4	11.3	12.4	12.6 ± 0.03	13.7	15.1	0	11
Carbohydrate (%)	55–79^a^	-	-	59.4	64.7	70.1	69.6 ± 0.1	75.1	79.1	4	10
**Antioxidants**											
Vitamin C (mg/d)	22.5	-	1,200	6.4	10.4	17.1	19.6 ± 0.2	25.2	36.2	68	0
Vitamin E (mg/d)		*6*	-	1	1.5	2.3	2.6 ± 0.03	3.3	4.5	-	-
**B vitamins**											
Thiamine (mg/d)	0.55	-	-	0.3	0.4	0.56	0.6 ± 0.004	0.76	0.97	48	-
Riboflavin (mg/d)	0.55	-	-	0.3	0.4	0.5	0.6 ± 0.01	0.69	0.9	58	-
Niacin (mg/d)	7	-	20	6.7	8.7	11.2	11.2 ± 0.1	14.3	17.6	11	4
Vitamin B6 (mg/d)	0.65	-	60	0.5	0.6	0.9	1.5 ± 0.03	1.8	3.3	27	0
Folate (DFE μg/d)	160	-	600	65	94	138	155.9 ± 1.5	197	268	61	0
Vitamin B12 (μg/d)	1.15	-	-	1.4	1.8	2.5	2.9 ± 0.03	3.5	4.9	5	-
**Bone-related nutrients**											
Calcium (mg/d)	440	-	2,500	132	173	232	250.8 ± 1.8	308	394	94	0
Phosphorus (mg/d)	405	-	4,000	351	440	556	583.5 ± 3.4	697	849	18	0
Magnesium (mg/d)	-	90	-	73	89	112	117.9 ± 0.7	140	171	-	-
Vitamin D (μg/d)	-	*5*	-	1.1	1.5	2.1	2.4 ± 0.02	3	4.2	-	-
**Other micronutrients**											
Vitamin A (μg RE/d)	271	-	1,700	117	168	243	304.9 ± 2.8	348	482	58	<1
Iron (mg/d)	8.2	-	-	3.5	4.6	6.2	6.8 ± 0.1	8.3	10.8	75	-
Zinc (mg/d)	3.4	-	23	2.6	3.3	4.3	5.2 ± 0.1	5.8	8.1	28	1
Sodium (mg/d)	-	*400*	-	329	497	748	834.2 ± 7.7	1,077	1,449	-	-
Potassium (mg/d)	-	*1,600*	-	486	596	745	783 ± 4.3	931	1,131	-	-
Selenium (μg/d)	15.4	-	280	39	49	64	67.9 ± 0.4	82	102	<1	0

1 Philippine Dietary Reference Intakes 2015.

^2^Adequate Intake (AI in italic text), Recommended Nutrient Intake (RNI in bold text).

**Table 3:** Usual energy and nutrient intakes from food and beverages for Filipino schoolchildren aged 10–12 years from 2013 NNS (*n* = 2,971)

Nutrients	Dietary reference intakes^1^			Mean/median intake percentiles						Inadequate/excessive reported intake	
	EAR/AMDR	AI/RNI	UL	10th	25th	Median	Mean ± SE	75th	90th	% <EAR/AMDR	% >AMDR/>UL
**Macronutrients**											
Energy intake (kcal/d)	1,967 (EER)			934	1,125	1,456	1,526.2 ± 9.5	1,818	2,207	-	-
Total fat (g/d)	-	-	-	10.2	16	25	28.8 ± 0.3	37.6	52.5	-	-
Saturated fat (g/d)	-	-	-	4	7	11	14.8 ± 0.3	17	28	-	-
Protein (g/d)	34.5	-	-	28.7	35.7	45.2	47.4 ± 0.3	56.6	69	21	-
Carbohydrate (g/d)	-	-	-	164	203	256	269.7 ± 1.7	321	393	-	-
Total sugars (g/d)	-	-	-	9.9	15.4	23.7	26.4 ± 0.3	34.3	46.2	-	-
Dietary fiber (g/d)	-	15–17	-	4.5	5.6	7.2	7.8 ± 0.1	9.2	11.8	-	-
**As percentage of total energy**											
Total fat (%)	15–30^a^	-	-	7.7	10.8	15.2	16 ± 0.1	20.2	25.2	49	3
Protein (%)	6–15^a^	-	-	10.7	11.47	12.4	12.6 ± 0.03	13.6	14.71	0	8
Carbohydrate (%)	55–79^a^	-	-	61.4	66.8	72.2	71.5 ± 0.1	76.9	80.6	3	16
**Antioxidants**											
Vitamin C (mg/d)	34.5	-	1,200	6.8	10.9	17.3	19.6 ± 0.2	25.6	35.4	89	0
Vitamin E (mg/d)		*8*	-	1.2	1.7	2.6	2.9 ± 0.03	3.8	5.3	-	-
**B vitamins**											
Thiamine (mg/d)	0.75	-	-	0.37	0.48	0.6	0.7 ± 0.01	0.9	1.1	63	-
Riboflavin (mg/d)	0.8	-	-	0.3	0.4	0.5	0.6 ± 0.01	0.7	0.97	81	-
Niacin (mg/d)	9.5	-	20	8.5	11	14	14.7 ± 0.1	17.7	21.6	16	15
Vitamin B6 (mg/d)	0.9	-	60	0.7	0.8	1.1	1.7 ± 0.03	2	3.8	35	0
Folate (DFE μg/d)	250	-	600	70	103	154	174.5 ± 1.8	223	307	81	0
Vitamin B12 (μg/d)	1.6	-	-	1.6	2.2	2.9	3.4 ± 0.03	4.2	5.7	9	-
**Bone-related nutrients**											
Calcium (mg/d)	440	-	2,500	157	200	260	278.3 ± 2	337	422	92	0
Phosphorus (mg/d)	1,055	-	4,000	431	542	688	718.3 ± 4.5	862	1,044	91	0
Magnesium (mg/d)	-	155	-	90	111	138	146 ± 0.9	173	211	-	-
Vitamin D (μg/d)	-	*5*	-	1.3	1.8	2.5	2.9 ± 0.03	3.5	4.6	-	-
**Other micronutrients**											
Vitamin A (μg RE/d)	369.5	-	1,700	143	198	285	345.9 ± 3.3	414	543	68	0
Iron (mg/d)	13.4	-	-	4.2	5.4	7.2	7.8 ± 0.1	9.4	12	90	-
Zinc (mg/d)	4.25	-	23	2.8	3.6	4.8	5.6 ± 0.1	6.8	9.5	40	0
Sodium (mg/d)	-	*500*	-	363	542	810	893.3 ± 8.7	1,156	1,536	-	-
Potassium (mg/d)	-	*2,000*	-	592	726	909	953.8 ± 5.8	1,134	1,376	-	-
Selenium (μg/d)	17.2	-	280	48	62	80	83.4 ± 0.6	101	124	<1	0

1 Philippine Dietary Reference Intakes 2015.

^2^Adequate Intake (AI in italic text), Recommended Nutrient Intake (RNI in bold text).

**Table 4:** Usual energy and nutrient intakes from food and beverages for Filipino adolescents aged 13−18 years from 2013 NNS (*n* = 5,447).

Nutrients	Dietary reference intakes^1^			Mean/median intake percentiles						Inadequate/excessive reported intake	
	EAR/AMDR	AI/RNI	UL	10th	25th	Median	Mean ± SE	75th	90th	% < EAR/AMDR	%>AMDR/>UL
**Macronutrients**											
Energy intake (kcal/d)	2,326 (EER)			1,076	1,334	1,680	1,756.5 ± 8	2,092	2,529	-	-
Total fat (g/d)	-	-	-	12	18	28	31.74 ± 0.25	41	56	-	-
Saturated fat (g/d)	-	-	-	5	8	12	14.1 ± 0.1	18	26	-	-
Protein (g/d)	54	-	-	35	44	55	58.16 ± 0.3	69	84	47	-
Carbohydrate (g/d)	-	-	-	186	233	296	312.25 ± 1.5	374	459	-	-
Total sugars (g/d)	-	-	-	10	15	23	26.16 ± 0.2	33	46	-	-
Dietary fiber (g/d)	-	20–23	-	5	6.3	8.1	8.69 ± 0.05	10.4	13	-	-
**As percentage of total energy**											
Total fat (%)	15–30^a^	-	-	8	11	15	15.59 ± 0.1	20	25	52	3
Protein (%)	6–15^a^	-	-	10.5	11.3	12.4	12.60 ± 0.04	13.7	14.9	0	10
Carbohydrate (%)	55–79^a^	-	-	62	67	73	71.78 ± 0.1	77	81	3	17
**Antioxidants**											
Vitamin C (mg/d)	54.5	-	1,800	8.9	13.8	21.5	24.28 ± 0.3	31.1	43.5	96	0
Vitamin E (mg/d)		*10.5*	-	1.3	1.8	2.7	2.9 ± 0.03	3.7	5	-	-
**B vitamins**											
Thiamine (mg/d)	1	-	-	0.5	0.6	0.8	0.83 ± 0.01	1	1.3	75	-
Riboflavin (mg/d)	1.05	-	-	0.39	0.49	0.63	0.68 ± 0.01	0.82	1.03	91	-
Niacin (mg/d)	12.5	-	30	10.9	13.8	17.7	18.44 ± 0.1	22.2	27	17	5
Vitamin B6 (mg/d)	1.15	-	80	0.8	1	1.3	1.6 ± 0.01	1.9	2.6	38	0
Folate (DFE μg/d)	330	-	800	80	112	163	179.8 ± 1.2	229	303	93	0
Vitamin B12 (μg/d)	21.5	-	-	1.8	2.4	3.3	3.6 ± 0.02	4.4	5.6	18	-
**Bone-related nutrients**											
Calcium (mg/d)	440	-	2,500	179	220	276	290.67 ± 1.3	345	421	92	0
Phosphorus (mg/d)	1,055	-	4,000	503	626	787	818.19 ± 3.6	975	1,173	82	0
Magnesium (mg/d)	-	495	-	105	129	160	167.8 ± 0.7	199	241	-	-
Vitamin D (μg/d)	-	*5*	-	1.2	1.8	2.6	3 ± 0.03	3.8	5.3	-	-
**Other micronutrients**											
Vitamin A (μg RE/d)	495	-	2,800	175	239	334	364.60 ± 2.3	456	593	81	0
Iron (mg/d)	14.2	-	-	4.9	6.2	8	8.52 ± 0.04	10.2	12.8	90	-
Zinc (mg/d)	5.4	-	34	3.2	4.1	5.4	6.5 ± 0.1	7.6	11	50	0
Sodium (mg/d)	-	*500*	-	405	580	833	896.85 ± 5.7	1,147	1,478	-	-
Potassium (mg/d)	-	*2,000*	-	685	842	1,052	1096.2 ± 4.8	1,301	1,564	-	-
Selenium (μg/d)	27.6	-	400	62	78	101	106.1 ± 0.5	128	157	<1	0

1 Philippine Dietary Reference Intakes 2015.

^2^Adequate Intake (AI in italic text), Recommended Nutrient Intake (RNI in bold text).

Inadequate intakes were found for all micronutrients except selenium in all age groups. Again, the extent of inadequacies increased with age. The highest inadequacies were for calcium (92–94%), iron (75–90%), vitamin C (68–96%), folate (61–93%), riboflavin (58–91%), and vitamin A (58–81%). In addition, among the schoolchildren aged 10–12 years and adolescents, a high prevalence of inadequacies was also found for thiamin (81–91%) and phosphorus (82–91%). The mean intakes of vitamin E, vitamin D, and potassium in all groups were far below the Adequate Intakes (AIs). Mean sodium intakes were, however, above the AIs (Tables 2–4).

### Intakes of energy and nutrients in relation to place of residence

In all age groups, the mean intakes of energy, total fat, saturated fat, protein, carbohydrate, and total sugar were significantly lower in rural than in urban areas. Mean fiber intakes appeared to be slightly higher in rural than in urban areas but were not statistically significant (Supplementary Table 1). In addition, in all groups, the mean intakes of most micronutrients were significantly lower in rural than in urban areas, including vitamin E, calcium, phosphorus, magnesium, vitamin A, zinc, iron, selenium, potassium, and sodium. Mean intakes of thiamin and riboflavin among schoolchildren aged 6–9 years and adolescents were significantly lower in rural areas compared with those in urban areas, while the mean intakes of these two nutrients among schoolchildren aged 10–12 years were significantly lower in urban areas compared with those in rural areas. On the contrary, in all age groups, the mean intakes of vitamin C and vitamin D, however, were significantly lower in urban than in rural areas (Table 5).

**Table 5:** Prevalence of inadequate nutrient intakes among schoolchildren and adolescents by place of residence

Nutrients	Prevalence of inadequacy (%) ± SE					
	6–9 years old		10–12 years old		13–18 years old	
	Rural (*n* = 2,060)	Urban (*n* = 1,534)	Rural (*n* = 1,764)	Urban (*n* = 885)	Rural (*n* = 3,109)	Urban (*n* = 2,337)
Protein (g/d)	56 ± 0.01*	37 ± 0.01	18 ± 0.02*	6 ± 0.01	33 ± 0.02*	11 ± 0.02
Total fat (%)	69 ± 0.01*	37 ± 0.02	55 ± 0.01*	21 ± 0.02	66 ± 0.02*	31 ± 0.02
Protein (%)	0	0	0	0	0	0
Carbohydrate (%)	1 ± 0.001*	4 ± 0.01	<1 ± 0.01*	6 ± 0.01	1 ± 0.004*	4 ± 0.01
Vitamin C (mg/d)	95 ± 0.02^NS^	96 ± 0.02	63 ± 0.02*	73 ± 0.04	88 ± 0.03^NS^	89 ± 0.04
Thiamine (mg/d)	82 ± 0.01*	67 ± 0.02	62 ± 0.01*	33 ± 0.02	75 ± 0.02*	51 ± 0.02
Riboflavin (mg/d)	94 ± 0.01*	89 ± 0.02	71 ± 0.01*	45 ± 0.02	89 ± 0.02*	71 ± 0.02
Niacin (mg/d)	26 ± 0.01*	8 ± 0.02	19 ± 0.01*	5 ± 0.01	24 ± 0.02*	7 ± 0.02
Vitamin B6 (mg/d)	42 ± 0.01*	33 ± 0.02	29 ± 0.02*	23 ± 0.02	42 ± 0.02*	26 ± 0.03
Vitamin B12 (mg/d)	18 ± 0.03^NS^	16 ± 0.03	6 ± 0.02*	4 ± 0.02	9 ± 0.03^NS^	8 ± 0.04
Folate DFE (μg/d)	91 ± 0.01*	96 ± 0.02	64 ± 4.1*	57 ± 0.02	80 ± 0.02*	84 ± 0.03
Calcium (mg/d)	93 ± 0.02^NS^	92 ± 0.02	95 ± 0.01*	93 ± 0.02	93 ± 0.02^NS^	91 ± 0.02
Phosphorus (mg/d)	85 ± 0.01*	80 ± 0.02	25 ± 0.01*	13 ± 0.02	93 ± 0.01*	88 ± 0.02
Vitamin A (μg RE/d)	80 ± 0.03*	83 ± 0.04	56 ± 0.01*	44 ± 0.02	68 ± 0.02*	60 ± 0.03
Zinc (mg/d)	59. ± 0.01*	38 ± 0.01	39 ± 0.01*	16 ± 0.02	50 ± 0.01*	27 ± 0.02
Iron (mg/d)	98 ± 0.01*	96 ± 0.01	87 ± 0.01*	76 ± 0.01	97 ± 0.01*	89 ± 0.01
Selenium (μg/d)	<1	0	0	0	0	0

* Significantly different from urban, *P* < 0.05, by using hypothesis testing to compare two population proportion with Bonferroni error correction.

NS Not significantly different.

From the above discussion, it can be stated that the prevalence of inadequate intakes of most macro- and micronutrients was higher in rural than in urban areas. Among schoolchildren aged 6–9 years, the prevalence of inadequate intakes of protein, total fat, thiamin, riboflavin, niacin, vitamin B6, calcium, phosphorus, vitamin A, zinc, and iron was significantly higher in rural than in urban areas, while the inadequacies of folate and vitamin A were higher in urban than in rural areas. Among schoolchildren aged 10–12 years, the prevalence of inadequate intakes of protein, total fat, thiamin, riboflavin, niacin, vitamin B6, folate, calcium, phosphorus, vitamin A, zinc, and iron was higher in rural areas compared with those in urban areas, whereas the inadequacy of vitamin C was significantly higher in urban areas compared with those in rural areas. Among adolescents, the inadequacy of protein, total fat, thiamin, riboflavin, niacin, vitamin B6, calcium, phosphorus, vitamin A, zinc, and iron was significantly higher in rural than in urban areas; however, the inadequacy of folate was significantly higher in urban than in rural areas (Table 5).

### Intakes of energy and nutrients in relation to wealth status

In all age groups, the mean intakes of energy and most macro- and micronutrients increased with wealth quintile moving up (Tables 6–8 and Supplementary Tables 1–4). Because of this, an overall higher prevalence of inadequate intakes was found among the poor and poorest schoolchildren and adolescents. For example, the prevalence of inadequate protein intake among the poorest was 25–69% compared to 1–20% among the richest. The prevalence of inadequate total fat intake as percentage of energy among the poorest was very high (77–84%), whereas those among the richest it was 1–20%. For most micronutrients, large to modest differences in the prevalence of inadequacies between wealth quintiles were found, including thiamin, riboflavin, niacin, calcium, iron, and zinc.

**Table 6:** Prevalence of inadequate nutrient intakes among schoolchildren aged 6–9 years from the 2013 NNS by wealth quintile

Nutrients	Prevalence of inadequacy ± SE (%)				
	Poorest (*n* = 1,021)	Poor (*n* = 791)	Middle (*n* = 649)	Rich (*n* = 562)	Richest (*n* = 468)
Protein (g/d)	25 ± 0.02^b,c,d,e^	15 ± 0.02^a,c,d,e^	7 ± 0.03^a,b,e^	5 ± 0.02^a,b,e^	1 ± 0.01^a,b,c,d^
Total fat (%)	77 ± 0.03^b,c,d,e^	48 ± 0.03^a,c,d,e^	27 ± 0.04^ab,d,e^	13 ± 0.04^a,b,c,e^	4 ± 0.04^a,b,c,d^
Protein (%)	0^NS^	0^NS^	0^NS^	0^NS^	0^NS^
Carbohydrate (%)	<1 ± 0.002^b,c,d,e^	1 ± 0.01^a,d,e^	1 ± 0.02^a,d,e^	3 ± 0.02^a,b,c,e^	12 ± 0.04^a,b,c,d^
Vitamin C (mg/d)	63 ± 0.04^b,c,d,e^	77 ± 0.04^a,c,d,e^	69 ± 0.06^a,b,e^	70 ± 0.06^a,b,e^	59 ± 0.04^a,b,c,d^
Thiamine (mg/d)	74 ± 0.02^b,c,d,e^	55 ± 0.02^a,c,d,e^	48 ± 0.03^a,b,d,e^	30 ± 0.04^a,b,c,e^	12 ± 0.06^a,b,c,d^
Riboflavin (mg/d)	85 ± 0.02^b,c,d,e^	69 ± 0.02^a,c,d,e^	55 ± 0.03^a,b,d,e^	40 ± 0.03^a,b,c,e^	18 ± 0.04^a,b,c,d^
Niacin (mg/d)	23 ± 0.02^b,c,d,e^	15 ± 0.02^a,c,d,e^	9 ± 0.02^a,b,d,e^	6 ± 0.02^a,b,c,e^	1 ± 0.01^a,b,c,d^
Vitamin B6 (mg/d)	31 ± 0.03^c,d,e^	35 ± 0.02^c,d,e^	20 ± 0.06^a,b,e^	18 ± 0.04^a,b,e^	7 ± 0.04^a,b,c,d^
Vitamin B12 (mg/d)	10 ± 0.04^c,d,e^	10 ± 0.03^c,d,e^	2 ± 0.03^a,b,d,e^	<1 ± 0.01^a,b,c^	<1 ± 0.02^a,b,c^
Folate DFE (μg/d)	67 ± 0.02^c,e^	65 ± 0.03^c,e^	59 ± 0.03^a,b,d,e^	65 ± 0.04^c,e^	42 ± 0.03^a,b,c,d^
Calcium (mg/d)	97 ± 0.01^d,e^	96 ± 0.02^d,e^	97 ± 0.02^d,e^	92 ± 0.03^a,b,c,e^	85 ± 0.04^a,b,c,d^
Phosphorus (mg/d)	34 ± 0.02^b,c,d,e^	22 ± 0.02^a,c,d,e^	15 ± 0.03^a,b,d,e^	11 ± 0.03^a,b,c,e^	3 ± 0.02^a,b,c,d^
Vitamin A (μg RE/d)	66 ± 0.03^c,d,e^	58 ± 0.03^a,c,d,e^	48 ± 0.03^a,b,d,e^	36 ± 0.03^a,b,c,e^	25 ± 0.06^a,b,c,d^
Zinc (mg/d)	50 ± 0.02^b,c,d,e^	33 ± 0.02^a,c,d,e^	29 ± 0.03^a,b,d,e^	13 ± 0.04^a,b,c,e^	1 ± 0.02^a,b,c,d^
Iron (mg/d)	92 ± 0.01^b,c,d,e^	86 ± 0.02^a,c,d,e^	80 ± 0.02^a,b,e^	80 ± 0.01^a,b,e^	66 ± 0.02^a,b,c,d^
Selenium (μg/d)	<1^NS^	0^NS^	0^NS^	0^NS^	0^NS^

Significantly different from ^a^poorest, ^b^poor, ^c^middle, ^d^rich, and ^e^richest, *P* < 0.05, by using hypothesis testing to compare two population proportion with Bonferroni error correction.

NS Not significantly different.

**Table 7:** Prevalence of inadequate nutrient intakes among schoolchildren aged 10–12 years from the 2013 NNS by wealth quintile

Nutrients	Prevalence of inadequacy ± SE (%)				
	Poorest (*n* = 885)	Poor (*n* = 696)	Middle (*n* = 519)	Rich (*n* = 418)	Richest (*n* = 380)
Protein (g/d)	43 ± 0.02^b,c,d,e^	27 ± 0.03^a,c,d,e^	19 ± 0.03^a,b,d,e^	7 ± 0.04^a,b,c,e^	2 ± 0.02^a,b,c,d^
Total fat (%)	84 ± 0.03^b,c,d,e^	63 ± 0.03^a,c,d,e^	40 ± 0.04^a,b,d,e^	20 ± 0.05^a,b,c,e^	6 ± 0.04^a,b,c,d^
Protein (%)	0^NS^	0^NS^	0^NS^	0^NS^	0^NS^
Carbohydrate (%)	<1 ± 0.01^c,d,e^	<1 ± 0.01^c,d,e^	1 ± 0.01^a,b,e^	1 ± 0.02^a,b,e^	3 ± 0.04^a,b,c,d^
Vitamin C (mg/d)	88 ± 0.04^b,c,e^	93 ± 0.05^a,c,e^	98 ± 0.06^a,b,d,e^	92 ± 0.07^c,e^	81 ± 0.1^a,b,c,d^
Thiamine (mg/d)	86 ± 0.02^b,c,d,e^	74 ± 0.03^a,c,d,e^	60 ± 0.03^a,b,d,e^	50 ± 0.03^a,b,c,e^	26 ± 0.04^a,b,c,d^
Riboflavin (mg/d)	97 ± 0.01^b,c,d,e^	89 ± 0.03^a,c,d,e^	81 ± 0.04^a,b,d,e^	72 ± 0.04^a,b,c,e^	42 ± 0.04^a,b,c,d^
Niacin (mg/d)	32 ± 0.02^b,c,d,e^	17 ± 0.03^a,d,e^	13 ± 0.03^a,d,e^	5 ± 0.03^a,b,c,e^	2 ± 0.02^a,b,c,d^
Vitamin B6 (mg/d)	47 ± 0.02^b,c,d,e^	39 ± 0.03^a,d,e^	35 ± 0.01^a,d,e^	21 ± 0.06^a.b.c.e^	12 ± 0.05^a,b,c,d^
Vitamin B12 (mg/d)	14 ± 0.05^c,d,e^	19 ± 0.04^c,d,e^	<1 ± 0.03^a,b^	<1 ± 0.03^a,b^	<1 ± 0.04^a,b^
Folate DFE (μg/d)	80 ± 0.02^d^	83 ± 0.04^NS^	82 ± 0.05^d^	87 ± 0.06^a,c,e^	80 ± 0.1^d^
Calcium (mg/d)	96 ± 0.02^d,e^	94 ± 0.02^e^	95 ± 0.03^d,e^	91 ± 0.04^a,c^	75 ± 0.05^a,b,c,d^
Phosphorus (mg/d)	95 ± 0.01^d,e^	92 ± 0.02^e^	94 ± 0.02^d,e^	88 ± 0.03^a,c^	81 ± 0.05^a,b,c^
Vitamin A (μg RE/d)	74 ± 0.04^d,e^	70 ± 0.03^d,e^	70 ± 0.1^d,e^	59 ± 0.1^a,b,c,e^	37 ± 0.06^a,b,c,d^
Zinc (mg/d)	64 ± 0.02^b,c,d,e^	48 ± 0.02^a,c,d,e^	7 ± 0.04^a,b,d^	20 ± 0.06^a,b,c,e^	5 ± 0.04^a,b,d^
Iron (mg/d)	98 ± 0.01^b,c,d,e^	95 ± 0.01^a,d^	93 ± 0.01^a,e^	91 ± 0.01^a,e^	84 ± 0.02^a.b,c,d^
Selenium (μg/d)	0^NS^	0^NS^	0^NS^	0^NS^	0^NS^

Significantly different from ^a^poorest, ^b^poor, ^c^middle, ^d^rich, and ^e^richest, *P* < 0.05, by using hypothesis testing to compare two population proportion with Bonferroni error correction.

NS Not significantly different.

**Table 8:** Prevalence of inadequate nutrient intakes among adolescents from the 2013 NNS by wealth quintile

Nutrients	Prevalence of inadequacy ± SE (%)				
	Poorest *(n* =1,333)	Poor (*n* = 1,203)	Middle (*n* = 1,082)	Rich (*n* = 903)	Richest (*n* = 781)
Protein (g/d)	69 ± 0.02^b,c,d,e^	56 ± 0.02^a,c,d,e^	43 ± 0.02^a,b,d,e^	35 ± 0.02^a,b,c,e^	20 ± 0.04^a,b,c,d^
Total fat (%)	84 ± 0.03^b,c,d,e^	70 ± 0.03^a,c,d,e^	57 ± 0.02^a,b,d,e^	30 ± 0.03^a,b,c,e^	9 ± 0.04^a,b,c,d^
Protein (%)	0^NS^	0^NS^	0^NS^	0^NS^	0^NS^
Carbohydrate (%)	<1 ± 0.03^c,d,e^	<1 ± 0.01^c,d,e^	1 ± 0.01^a,b,d,e^	3 ± 0.02^a,b,c,e^	9 ± 0.03^a,b,c,d^
Vitamin C (mg/d)	93 ± 0.03^b,c,e^	98 ± 0.02^a,d^	97 ± 0.03^a^	95 ± 0.03^b,e^	98 ± 0.05^a,d^
Thiamine (mg/d)	90 ± 0.02^b,c,d,e^	86 ± 0.03^a,c,d,e^	75 ± 0.03^a,b,d,e^	64 ± 0.03^a,b,c,e^	52 ± 0.03^a,b,c,d^
Riboflavin (mg/d)	98 ± 0.01^b,c,d,e^	96 ± 0.01^a,c,d,e^	92 ± 0.03^a,b,d,e^	88 ± 0.04^a,b,c,e^	81 ± 0.06^a,b,c,d^
Niacin (mg/d)	35 ± 0.02^b,c,d,e^	22 ± 0.02^a,c,d,e^	12 ± 0.03^a,b,e^	9 ± 0.03^a,b,e^	2 ± 0.02^a,b,c,d^
Vitamin B6 (mg/d)	52 ± 0.02^c,d,e^	47 ± 0.02^c,d,e^	29 ± 0.04^a,b,e^	30 ± 0.03^a,b,e^	8 ± 0.07^a,b,c,d^
Vitamin B12 (mg/d)	21 ± 0.04^e^	24 ± 0.03^d,e^	21 ± 0.06^e^	17 ± 0.04^b,e^	3 ± 0.06^a,b,c,d^
Folate DFE (μg/d)	87 ± 0.02^b,c,d,e^	94 ± 0.02^a,d^	94 ± 0.02^a,d^	98 ± 0.02^a,b,c,e^	95 ± 0.03^a,d^
Calcium (mg/d)	94 ± 0.04^e^	96 ± 0.02^c,d,e^	93 ± 0.03^b,e^	91 ± 0.03^b,e^	87 ± 0.05^a,b,c,d^
Phosphorus (mg/d)	90 ± 0.02^b,c,d,e^	86 ± 0.02^a,c,d,e^	80 ± 0.02^a,b^	79 ± 0.03^a,b^	76 ± 0.03^a,b^
Vitamin A (μg RE/d)	82 ± 0.04^NS^	86 ± 0.06^d,e^	83 ± 0.07	79 ± 0.05^b^	78 ± 0.09^b^
Zinc (mg/d)	99 ± 0.01^d,e^	99 ± 0.01^d,e^	98 ± 0.01^e^	96 ± 0.01^a,b^	95 ± 0.01^a,b,c^
Iron (mg/d)	70 ± 0.02^b,c,d,e^	57 ± 0.02^a,c,d,e^	50 ± 0.02^a,b,d,e^	33 ± 0.03^a,b,c,e^	19 ± 0.04^a,b,c,d^
Selenium (μg/d)	1^b,c,d^	<1^NS^	0^NS^	0^NS^	0^NS^

Significantly different from ^a^poorest, ^b^poor, ^c^middle, ^d^rich, and ^e^richest, *P* < 0.05, by using hypothesis testing to compare two population proportion with Bonferroni error correction.

NS Not significantly different.

However, the above patterns were not found for vitamin C and folate. The mean vitamin C intake among the poorest adolescents was higher than that in other wealth quintiles; hence, the prevalence of inadequacy was lower (Table 8 and Supplementary Tables 2–4). However, it is worth mentioning that in any case the prevalence of inadequate vitamin C intake among adolescents across all wealth quintiles were high (93 to 98%) (Table 8). Furthermore, the mean folate intake among the poorest adolescents was higher than that from other wealth quintiles, hence a lower prevalence of inadequacy (Table 8 and Supplementary Table 4).

## Discussion

### Inadequate energy and nutrient intakes

This study provides estimates of usual energy and nutrients from the food and beverages consumed by a representative sample of Filipino schoolchildren and adolescents and the prevalence of inadequacy. The results indicate marked inadequacies in the intakes of energy, fat, and most micronutrients. The high inadequacies of calcium, iron, vitamins A and C, and folate should be a cause of concern, as these are the key nutrients required for growth and development in this population (4). The prevalence of inadequacies was worse among the adolescents, the poor segments of all age groups, and those living in rural areas. The data also imply that despite the steady economic growth of the country over the past few decades (23), large shortfall in the diets of Filipinos is still a pressing issue. Indeed, our findings are supported by recent reports, which revealed that about seven out of 10 Filipino households are still experiencing food insecurity (24), and the current diets of a majority of Filipinos are monotonous, comprising mostly of cereals, in fact, predominately refined rice (14, 25).

The results of this study are in conformity with studies conducted in other developing countries, in which the researchers found that the intakes of most micronutrients among children were suboptimal and the diets consumed by the children and adolescents in those developing countries were generally inadequate for energy and fats (2, 26).

### Place of residence and nutrient intakes

We found the proportions of schoolchildren and adolescents not meeting the recommendations for energy and nutrients were higher in rural areas than those in urban areas. This is in line with the findings of studies conducted in both developing and developed countries (8, 26). The reasons are likely to be multifactorial. First, these inequalities might be driven by poverty. Among the Filipino households residing in rural areas, where the population is generally engaged in agriculture, more than half (58.4%) were classified as poor (27). Many Filipinos in rural areas suffer from lack of food or poor diets because of inadequate access to food or food rich in nutrients (28). This is indeed the situation observed in many other low- and middle-income countries, where children living in rural areas experience lower dietary diversity and lower intakes of important nutrients, whereas those in urban areas have higher intakes of calories, protein, total fat, and micronutrients (10). Second, unavailability of healthy foods could be another reason behind the differences between rural and urban areas. Food variety is often much greater in urban communities since food markets are better supplied and more accessible, whereas this is not the case in rural areas (29). In addition, a low income not only restricts the ability of households to buy foods rich in nutrients but also limits their access to food retailers. Few of the poor families have access to a private vehicle; therefore, they have to shop in local markets, where food can be more expensive than in supermarkets (30).

Unlike other nutrients, the prevalence of inadequacy for vitamin C and folate was lower in rural than in urban groups. A lower prevalence of inadequacy for folate and a higher fiber intake was also observed among the rural groups. This may be a result from a higher vegetable intake among rural households as reported by the 2013 NNS (14). In a review on global fruit and vegetable consumption, a higher vegetable consumption was reported in rural versus urban areas, but the overall fruit and vegetable consumption in Philippine population was considered low (31).

### Wealth status and nutrient intakes

The influence of SES on dietary intakes has been reported in many studies around the world (7, 8, 32–34). In our study, the effect of wealth status on dietary intake was illustrated by the clear income gradient with respect to energy and nutrient intakes in all age groups. The mean intakes of energy and most macro- and micronutrients increased with increasing wealth quintile. Hence, schoolchildren and adolescents of the poorest quintile had the highest and the richest quintile had the lowest prevalence of inadequacies. This finding is indeed supported by the higher prevalence of stunting and wasting among the poor segments of the population reported by the 2013 NNS (12).

The low mean intakes of all vitamins and most minerals, as well as the large proportion of poor schoolchildren and adolescents with these nutrients below the EAR, indicate poor food variety. The diets of the lower wealth quintile groups comprised of mainly rice, with little intake of meat, fruit, vegetables, and milk (14), and hence very low in essential nutrients. The low fat intake among the schoolchildren and adolescents with low wealth quintile compared with those with high wealth quintile indicated a low fat consumption among the poor. Coconut oil, which is the main source of cooking oil, may not always be available to families with low wealth quintile due to lack of money (14).

It is worth mentioning that unlike most nutrients, the mean intake of calcium and the prevalence of inadequacy between rural and urban areas and between wealth quintiles were small. The prevalence of inadequate calcium intakes was high in all groups (75–97%). Low calcium intake had been reported in Filipino children before (35). It is well known that milk and dairy products are the primary source of calcium, and the low intake of calcium can be attributed to the high price of milk and dairy products (30) and social isolation, which makes access to supermarkets more difficult. Another nutrient is iron. Iron inadequacy increased as age increased, with a slight increase between 10–12-year-olds and 13–18-year-olds. Most importantly, the inadequacy was consistently high among all quintiles in these two age groups. The possible reason for this could be that the diets consumed lack iron but EARs are set high for these age groups in order to meet their rapid growth (21).

### Limitation of the study

This study has a number of limitations. First, the study did not take into account the potential intake of vitamin and mineral supplements of the children that were additional to their diets; therefore, this could result in a potential underestimation of micronutrient intakes in this study population. Second, as with all national surveys regarding food and beverage intake of children, the study results rely on self-reported dietary intake by the parents and children; hence, there lies the risk of overestimation and underestimation of the food intake. Another limitation of this study was that the updated Philippine food composition tables used in this study were expanded by adopting some data from the food composition tables of different countries. Food composition from other countries may not reflect the foods in Philippine population, especially for the mixed dishes prepared at home.

## Conclusions

This study evaluated the usual energy and nutrient intakes of Filipino schoolchildren and adolescents and demonstrated that the intakes of energy, fat, and most key micronutrients were markedly inadequate. The inadequacies were more common among the poor and those living in rural areas. The results reinforce the need to focus on effective implementation of programs among the populations that are at high risk of nutrient deficiencies. The findings of this study could be used by government agencies, non-profit organizations, local government units, and other policy makers in the Philippines for developing programs, intervention policies, and advocacy initiatives addressing the needs of these age groups specifically. In addition, the knowledge from this study will also enable food industry to formulate food products that could target the nutritional needs of this population. Further studies to understand food sources energy and nutrients among the schoolchildren and adolescents are currently underway.

## Supplementary Material

Inadequate nutrient intakes in Filipino schoolchildren and adolescents are common among those from rural areas and poor families
