# Supplementary material for: Inadequate nutrient intakes in Filipino schoolchildren and adolescents are common among those from rural areas and poor families
Source: Food Nutr Res. 2019 May 16;63:10.29219/fnr.v63.3435. doi: 10.29219/fnr.v63.3435 (PMC6525598; doi:10.29219/fnr.v63.3435)
Supplement: Inadequate nutrient intakes in Filipino schoolchildren and adolescents are common among those from rural areas and poor families [file FNR-63-3435-s001.docx]

Supplementary Table 1. Mean usual energy and nutrient intakes among Filipino schoolchildren and adolescents by place of residence

|  | **Mean ± SE** | | | | | |
| --- | --- | --- | --- | --- | --- | --- |
|  | **6-9 years old** | | **10-12 years old** | | **13-18 years old** | |
| **Nutrients** | **Rural (n=2060)** | **Urban (n=1534)** | **Rural (n=1764)** | **Urban (n=885)** | **Rural (n=3109)** | **Urban (n=2337)** |
| Energy (kcal/d) | 1144.7 ± 8.9* | 1336.7 ± 10.6 | 1405.1 ± 11.7* | 1653.4 ± 14.5 | 1657.2 ± 10.43* | 1849.5 ± 11.7 |
| ***Macronutrients*** |  |  |  |  |  |  |
| Total fat (g/d) | 20.2 ± 0.3* | 31.7 ± 0.4 | 25.1 ± 0.3* | 36 ± 0.5 | 24.82 ± 0.3* | 38.24 ± 0.36 |
| Saturated fat (g/d) | 9.7 ± 0.2* | 15.7 ± 0.3 | 11.2 ± 0.3* | 18.5 ± 0.5 | 11.2 ± 0.2* | 16.8 ± 0.2 |
| Protein (g/d) | 35.7 ± 0.3* | 41.7 ± 0.3 | 43.2 ± 0.4* | 51.9 ± 0.4 | 54.15 ± 0.3* | 61.90 ± 7.62 |
| Carbohydrate (g/d) | 205.1 ± 1.6* | 222.2 ± 1.9 | 259.2 ± 2.3* | 280.8 ± 2.5 | 306.87 ± 2* | 317.41 ± 2.24 |
| Total sugars (g/d) | 22.6 ± 0.3* | 31.7 ± 0.5 | 23.9 ± 0.4* | 29.5 ± 0.4 | 23.42 ± 0.2* | 28.99 ± 0.37 |
| Dietary fiber (g/d) | 6.3 ± 0.1^NS^ | 6.22 ± 0.1 | 7.9 ± 0.1^NS^ | 7.7 ± 0.2 | 9.17 ± 0.1* | 8.22 ± 0.05 |
| ***As percentage of total energy*** |  |  |  |  |  |  |
| Total Fat (%) | 14.9 ± 0.1* | 20.6 ± 0.2 | 13.3 ± 0.1* | 18.8 ± 0.2 | 12.82 ± 0.1* | 18.18 ± 0.13 |
| Protein (%) | 12.7 ± 0.1^NS^ | 12.6 ± 0.04 | 12.5 ± 0.04* | 12.7 ± 0.04 | 12.41 ± 0.03* | 12.77 ± 0.04 |
| Carbohydrate (%) | 72.5 ± 0.2* | 66.8 ± 0.2 | 74.3 ± 0.2* | 68.5 ± 0.2 | 74.73 ± 0.1* | 68.99 ± 0.15 |
| ***Antioxidants*** |  |  |  |  |  |  |
| Vitamin C (mg/d) | 21.1 ± 0.3* | 18.2 ± 0.3 | 20 ± 0.3* | 19.2 ± 0.3 | 26.22 ± 0.3* | 22.34 ± 0.30 |
| Vitamin E (mg/d) | 2.4 ± 0.04* | 2.8 ± 0.03 | 2.8 ± 0.04* | 3.2 ± 0.1 | 2.9 ± 0.03* | 3 ± 0.02 |
| ***B vitamins*** |  |  |  |  |  |  |
| Thiamine (mg/d) | 0.5 ± 0.01* | 0.7 ± 0.01 | 0.7 ± 0.01* | 0.6 ± 0.01 | 0.75 ± 0.01* | 0.90 ± 0.01 |
| Riboflavin (mg/d) | 0.5 ± 0.01* | 0.6 ± 0.01 | 0.6 ± 0.01* | 0.5 ± 0.01 | 0.61 ± 0.01* | 0.74 ± 0.01 |
| Niacin (mg/d) | 10.9 ± 0.1* | 12.7 ± 0.1 | 14.7 ± 0.1* | 13.3 ± 0.1 | 17.3 ± 0.1* | 19.58 ± 0.12 |
| Vitamin B6 (mg/d) | 1.6 ± 0.04^NS^ | 1.5 ± 0.04 | 1.2 ± 0.02* | 1.4 ± 0.02 | 1.5 ± 0.01* | 1.6 ± 0.01 |
| Vitamin B12 (mg/d) | 4.1 ± 0.04* | 2.7 ± 0.03 | 3.6 ± 0.1* | 3.2 ± 0.04 | 3.6 ± 0.03* | 3.5 ± 0.03 |
| Folate DFE (µg/d) | 150.6 ± 4.1* | 159.9 ± 1.8 | 175 ± 2.7^NS^ | 175.1 ± 2.4 | 183.7 ± 1.8* | 175 ± 1.6 |
| ***Bone-related nutrients*** |  |  |  |  |  |  |
| Calcium (mg/d) | 235.2 ± 2.4* | 266.7 ± 2.8 | 265.1 ± 2.6* | 292.3 ± 5.1 | 287.83 ± 1.74* | 293.39 ± 2.09 |
| Phosphorus (mg/d) | 545.5 ± 4.3* | 619.9 ± 5.1 | 665.3 ± 5.8* | 773.9 ± 4.5 | 781.47 ± 4.8* | 852.82 ± 5.33 |
| Magnesium (mg/d) | 113 ± 0.9* | 122.5 ± 1.1 | 140.5 ± 1.2* | 151.8 ± 1.5 | 166.9 ± 1NS | 168.8 ± 1.1 |
| Vitamin D (mg/d) | 2.6 ± 0.04* | 2.3 ± 0.03 | 2.8 ± 0.04* | 2.8 ± 0.03 | 3.2 ± 0.04* | 2.9 ± 0.02 |
| ***Other micronutrients*** |  |  |  |  |  |  |
| Vitamin A (µg RE/d) | 285 ± 3.6* | 323.1 ± 4.2 | 328.6 ± 4.2* | 360.1 ± 5.1 | 369.22 ± 3.4* | 356.89 ± 3.36 |
| Zinc (mg/d) | 4.7 ± 0.1* | 5.5 ±0.1 | 4.9 ± 0.1* | 6.2 ± 0.1 | 5.8 ± 0.1* | 7 ± 0.1 |
| Iron (mg/d) | 5.8 ± 0.03* | 7.7 ± 0.1 | 6.8 ± 0.1* | 8.7 ± 0.1 | 7.74 ± 0.1* | 9.24 ± 0.06 |
| Sodium (mg/d) | 698.9 ± 9.2* | 964.5 ± 11.7 | 765.1 ± 10.5* | 1029.2 ± 13.7 | 762.93 ± 6.8* | 1024.2 ± 9 |
| Selenium (µg/d) | 60.5 ± 1.2* | 74.9 ± 0.6 | 74.5 ± 0.7* | 92.8 ± 0.8 | 96.6 ± 0.7* | 114.9 ± 0.7 |
| Potassium (mg/d) | 757.7 ± 0.1* | 807.9 ± 6.8 | 910.5 ± 7.3* | 1000 ± 9.4 | 1070.7 ± 6.4* | 1120.5 ± 7.3 |

*Significantly different from urban, P < 0.05 by using hypothesis testing to compare two population proportion with Bonferroni Error Correction. ^NS^ not significantly different.

Supplementary Table 2. Mean usual energy and nutrient intakes among schoolchildren aged 6-9 years from the 2013 NNS by wealth quintile

|  | **Mean ± SE** | | | | |
| --- | --- | --- | --- | --- | --- |
| **Nutrients** | **Poorest (n=1021)** | **Poor (n=791)** | **Middle (n=649)** | **Rich (n=562)** | **Richest (n=468)** |
| Energy (kcal/d) | 1066.1 ± 12.4^b,c,d,e^ | 1206.7 ± 13.8^a,d,e^ | 1207.9 ± 14.2^a,d,e^ | 1328.9 ± 17.7^a,b,c,e^ | 1491.4 ± 18.7^a,b,c,d^* |
| ***Macronutrients*** |  |  |  |  |  |
| Total fat (g/d) | 14.5 ± 0.3^b,c,d,e^ | 22.4 ± 0.4^a,c,d,e^ | 25.7 ± 0.4^a,b,d,e^ | 31.3 ± 0.5^a,b,c,e^ | 41.4 ± 0.7^a,b,c,d^* |
| Saturated fat (g/d) | 7.3 ± 0.2^b,c,d,e^ | 10.3 ± 0.2^a,c,d,e^ | 12.6 ± 0.4^a,b,d,e^ | 15.7 ± 0.4^a,b,c,e^ | 18.7 ± 0.5^a,b,c,d^* |
| Protein (g/d) | 31.7 ± 0.3^b,c,d,e^ | 37.1 ± 0.5^a,d,e^ | 37.8 ± 0.4^a,d,e^ | 41.7 ± 0.5^a,b,c,e^ | 48.8 ± 0.6^a,b,c,d^* |
| Carbohydrate (g/d) | 201.1 ± 2.4^b,d,e^ | 215.8 ± 2.6^a,e^ | 206.6 ± 2.5^d,e^ | 220 ± 3.2^a,c,e^ | 233.2 ± 3.4^a,b,c,d^* |
| Total sugars (g/d) | 18.7 ± 0.4^b,c,d,e^ | 24.7 ± 0.5^a,c,d,e^ | 26.3 ± 0.6^a,b,d,e^ | 32.5 ± 0.8^a,b,c,e^ | 37.6 ± 0.8^a,b,c,d^* |
| Dietary fiber (g/d) | 6.4 ± 0.1^d,e^ | 6.2 ± 0.1^e^ | 6.2 ± 0.1^e^ | 6 ± 0.1^a,e^ | 6.7 ± 0.1^a,b,c,d^* |
| ***As percentage of total energy*** |  |  |  |  |  |
| Total Fat (%) | 11.7 ± 0.2^b,c,d,e^ | 15.7 ± 0.2^a,c,d,e^ | 18.5 ± 0.2^a,b,d,e^ | 21 ± 0.2^a,b,c,e^ | 24.4 ± 0.3^a,b,c,d^* |
| Protein (%) | 12.4 ± 0.1^c,d,e^ | 12.3 ± 0.1^c,d,e^ | 12.7 ± 0.04^a,b,e^ | 12.7 ± 0.1^a,b,e^ | 13.2 ± 0.1^a,b,c,d^* |
| Carbohydrate (%) | 75.9 ± 0.2^b,c,d,e^ | 72 ± 0.2^a,c,d,e^ | 68.9 ± 0.2^a,b,d,e^ | 66.3 ± 0.2^a,b,c,e^ | 62.4 ± 0.3^a,b,c,d^* |
| ***Antioxidants*** |  |  |  |  |  |
| Vitamin C (mg/d) | 21.1 ± 0.4^b,c,d,e^ | 16.4 ± 0.4^a,c,d,e^ | 19 ± 0.5^a,b,e^ | 19 ± 0.5^a,b,e^ | 23 ± 0.3^a,b,c,d^* |
| Vitamin E (mg/d) | 2.3 ± 0.1^c,d,e^ | 2.3 ± 0.04^d,e^ | 2.3 ± 0.04^a,d,e^ | 2.8 ± 0.1^a,b,c,e^ | 3.2 ± 0.1^a,b,c,d^* |
| ***B vitamins*** |  |  |  |  |  |
| Thiamine (mg/d) | 0.5 ± 0.01^b,c,d,e^ | 0.6 ± 0.01^a,c,d,e^ | 0.6 ± 0.01^a,b,d,e^ | 0.7 ± 0.01^a,b,c,e^ | 0.8 ± 0.01^a,b,c,d^* |
| Riboflavin (mg/d) | 0.4 ± 0.01^b,c,d,e^ | 0.5 ± 0.01^a,c,d,e^ | 0.6 ± 0.01^a,b,d,e^ | 0.7 ± 0.01^a,b,c,e^ | 0.8 ± 0.01^a,b,c,d^* |
| Niacin (mg/d) | 10 ± 0.1^b,c,d,e^ | 11.4 ± 0.2^a,d,e^ | 11.4 ± 0.1^a,d,e^ | 12.8 ± 0.2^a,b,c,e^ | 15.4 ± 0.2^a,b,c,d^* |
| Vitamin B6 (mg/d) | 1.7 ± 0.2^c,e^ | 1.4 ± 0.2^c,e^ | 1.7 ± 0.1^a,b,d,e^ | 1.5 ± 0.1^c,e^ | 2.2 ± 0.2^a,b,c,d^* |
| Vitamin B12 (mg/d) | 2.8 ± 0.1^d,e^ | 2.9 ± 0.1^d^ | 2.6 ± 0.1^d,e^ | 2.9 ± 0.04^a,b,c^ | 2.9 ± 0.04^a,c^* |
| Folate DFE (µg/d) | 143.9 ± 3.2^c,d,e^ | 146.9 ± 2.8^c,e^ | 160.5 ± 3.2^a,b,e^ | 147 ± 2.3^a,e^ | 191.8 ± 4.1^a,b,c,d^* |
| ***Bone-related nutrients*** |  |  |  |  |  |
| Calcium (mg/d) | 204.5 ± 3^b,c,d,e^ | 239.3 ± 3.6^a,d,e^ | 235.7 ± 3.4^a,d,e^ | 276.6 ± 4.7^a,b,c,e^ | 322.3 ± 5.4^a,b,c,d^* |
| Phosphorus (mg/d) | 500.6 ± 5.8^b,c,d,e^ | 557.1 ± 7^a,d,e^ | 569.2 ± 6.4^a,d,e^ | 623.9 ± 8.2^a,b,c,e^ | 710.8 ± 9.2^a,b,c,d^* |
| Magnesium (mg/d) | 110.2 ± 1.3^b,c,e^ | 115.9 ± 1.4^a,e^ | 111.6 ± 1.3^d,e^ | 118.5 ± 1.6^a,c,e^ | 139.4 ± 1.8^a,b,c,d^* |
| Vitamin D (mg/d) | 2.4 ± 0.1^b,c,d,e^ | 2.4 ± 0.1^a,d,e^ | 2.4 ± 0.03^a^ | 2.5 ± 0.04^a,b^ | 2.5 ± 0.04^a,b^* |
| ***Other micronutrients*** |  |  |  |  |  |
| Vitamin A (µg RE/d) | 245.7 ± 4.7^b,c,d,e^ | 274.9 ± 5.3^a,c,d,e^ | 303.6 ± 5.7^a,b,d,e^ | 350.3 ± 7.1^a,b,c,e^ | 375.6 ± 6.9^a,b,c,d^* |
| Zinc (mg/d) | 4.5 ± 0.2^b,c,d,e^ | 4.6 ± 0.1^a,d,e^ | 4.7 ± 0.1^a,d,e^ | 5.5 ± 0.1^a,b,c,e^ | 6.5 ± 0.1^a,b,c,d^* |
| Iron (mg/d) | 5 ± 0.1^b,c,d,e^ | 6.3 ± 0.1^a,c,d,e^ | 6.9 ± 0.1^a,b,d,e^ | 7.4 ± 0.1^a,b,c,e^ | 9.14 ± 0.2^a,b,c,d^* |
| Sodium (mg/d) | 620.4 ± 13.6^b,c,d,e^ | 754.9 ± 11.8^a,c,d,e^ | 828.3 ± 17.4^a,b,d,e^ | 943.7 ± 13.6^a,b,c,e^ | 1143.3 ± 24.1^a,b,c,d^* |
| Selenium (µg/d) | 54.5 ± 0.7^b,c,d,e^ | 64.8 ± 0.9^a,c,d,e^ | 66.9 ± 0.8^a,b,d,e^ | 74.3 ± 1.1^a,b,c,e^ | 85.5 ± 1.2^a,b,c,d^* |
| Potassium (mg/d) | 710.3 ± 7.7^b,c,d,e^ | 758.6 ± 9.8^a,d,e^ | 736.9 ± 7.5^a,d,e^ | 798.6 ± 11.8^a,b,c,e^ | 959.3 ± 11.7^a,b,c,d^* |
|  |  |  |  |  |  |

Significantly different from ^a^ poorest, ^b^ poor, ^c^ middle, ^d^ rich and ^e^ richest, P < 0.05 by using hypothesis testing to compare two population proportion with Bonferroni Error Correction. ^NS^ not significantly different.

Supplementary Table 3. Mean usual energy and nutrient intakes and among schoolchildren aged 10-12 year from the 2013 NNS by wealth quintile

|  | | **Mean ± SE^1^** | | | | | | | | |
| --- | --- | --- | --- | --- | --- | --- | --- | --- | --- | --- |
| **Nutrients** | | **Poorest (n=885)** | | **Poor (n=696)** | | **Middle (n=519)** | | **Rich (n=418)** | | **Richest (n=380)** |
| Energy (kcal/d) | 1286.2 ± 15.9^b,c,d,e^ | | 1467.4 ± 17.7^a,c,d,e^ | | 1532.3 ± 20.8^a,b,d,e^ | | 1651.6 ± 25.5^a,b,c,e^ | | 1862 ± 21.9^a,b,c,d^* | |
| ***Macronutrients*** |  | |  | |  | |  | |  | |
| Total fat (g/d) | 15.7 ± 0.3^b,c,d,e^ | | 23.6 ± 0.5^a,c,d,e^ | | 29.2 ± 0.6^a,b,d,e^ | | 36.5 ± 0.7^a,b,c,e^ | | 49.3 ± 0.8^a,b,c,d^* | |
| Saturated fat (g/d) | 8 ± 0.3^b,c,d,e^ | | 11 ± 0.3^a,c,d,e^ | | 14.6 ± 0.4^a,b,d,e^ | | 22.5 ± 0.9^a,b,c,e^ | | 23.7 ± 0.5^a,b,c,d^* | |
| Protein (g/d) | 38.3 ± 0.4^b,c,d,e^ | | 44.6 ± 0.6^a,c,d,e^ | | 46.9 ± 0.6^a,b,d,e^ | | 53.2 ± 0.7^a,b,c,e^ | | 60.7 ± 0.8^a,b,c,d^* | |
| Carbohydrate (g/d) | 246.9 ± 5.1^b,c,d,e^ | | 271.1 ± 3.6^a,e^ | | 270.1 ± 5.1^a,e^ | | 277 ± 4.7^a,e^ | | 296.8 ± 3.8^a,b,c,d^* | |
| Total sugars (g/d) | 18.8 ± 0.4^b,c,d,e^ | | 23 ± 0.4^a,c,d,e^ | | 26 ± 0.6^a,b,d,e^ | | 30.2 ± 0.9^a,b,c,e^ | | 39.6 ± 0.8^a,b,c,d^* | |
| Dietary fiber (g/d) | 7.7 ± 0.1^d,e^ | | 7.7 ± 0.1^e^ | | 7.8 ± 0.1^d,e^ | | 7.2 ± 0.1^a,c,e^ | | 8.4 ± 0.1^a,b,c,d^* | |
| ***As percentage of total energy*** |  | |  | |  | |  | |  | |
| Total Fat (%) | 10.5 ± 0.2^b,c,d,e^ | | 13.9 ± 0.2^a,c,d,e^ | | 16.8 ± 0.2^a,b,d,e^ | | 19.6 ± 0.3^a,b,c,e^ | | 23.2 ± 0.3^a,b,c,d^* | |
| Protein (%) | 12.3 ± 0.1^c,d,e^ | | 12.3 ± 0.1^c,d,e^ | | 12.5 ± 0.1^a,b,d,e^ | | 15.1 ± 0.1^a,b,c^ | | 15.1 ± 0.1^a,b,c^* | |
| Carbohydrate (%) | 77.3 ± 0.2^b,c,d,e^ | | 73.9 ± 0.2^a,c,d,e^ | | 70.8 ± 0.3^a,b,d,e^ | | 67.3 ± 0.3^a,b,c,e^ | | 63.7 ± 0.3^a,b,c,d^* | |
| ***Antioxidants*** |  | |  | |  | |  | |  | |
| Vitamin C (mg/d) | 19.4 ± 0.4^b,e^ | | 17.2 ± 0.4^a,c,d,e^ | | 17.7 ± 0.3^b,e^ | | 18.6 ± 0.5^b,e^ | | 25.6 ± 0.6^a,b,c,d^* | |
| Vitamin E (mg/d) | 2.8 ± 0.1^d,e^ | | 2.6 ± 0.1^c,d,e^ | | 2.8 ± 0.04^c,d,e^ | | 5.1 ± 0.1^a,b,d,e^ | | 3.9 ± 0.04^a,b,c,d^* | |
| ***B vitamins*** |  | |  | |  | |  | |  | |
| Thiamine (mg/d) | 0.5 ± 0.01^b,c,d,e^ | | 0.6 ± 0.01^a,c,d,e^ | | 0.7 ± 0.01^a,b,d,e^ | | 0.8 ± 0.01^a,b,c,e^ | | 1 ± 0.01^a,b,c,d^* | |
| Riboflavin (mg/d) | 0.4 ± 0.01^b,c,d,e^ | | 0.5 ± 0.01^a,c,d,e^ | | 0.6 ± 0.01^a,b,d,e^ | | 0.7 ± 0.01^a,b,c,e^ | | 0.9 ± 0.01^a,b,c,d^* | |
| Niacin (mg/d) | 12 ± 0.2^b,c,d,e^ | | 14 ± 0.2^a,c,d,e^ | | 14.7 ± 0.2^a,b,d,e^ | | 16 ± 0.2^a,b,c,e^ | | 18.3 ± 0.3^a,b,c,d^* | |
| Vitamin B6 (mg/d) | 1.1 ± 0.03^c,d,e^ | | 1.2 ± 0.02^c,d,e^ | | 1.6 ± 0.1^a,b,e^ | | 1.4 ± 0.03^a,b,e^ | | 1.5 ± 0.04^a,b,c,d^* | |
| Vitamin B12 (mg/d) | 3.3 ± 0.1^c,d,e^ | | 3.2 ± 0.1^c,d,e^ | | 3.4 ± 0.04^a,b^ | | 3.6 ± 0.1^a,b^ | | 3.7 ± 0.1^a,b^* | |
| Folate DFE (µg/d) | 167.6 ± 4.2^c,e^ | | 165.9 ± 3.9^c,e^ | | 181.4 ± 3.7^a,b,d,e^ | | 165.9 ± 3.4^c,e^ | | 196.1 ± 3.5^a,b,c,d^* | |
| ***Bone-related nutrients*** |  | |  | |  | |  | |  | |
| Calcium (mg/d) | 242.6 ± 3.2^b,c,d,e^ | | 255.7 ± 0.9^a,d,e^ | | 265.1 ± 3.7^a,d,e^ | | 296.7 ± 5.8^a,b,c,e^ | | 356.3 ± 6.7^a,b,c,d^* | |
| Phosphorus (mg/d) | 611 ± 7.7^b,c,d,e^ | | 689.7 ± 9^a,d,e^ | | 706 ± 9.2^a,d,e^ | | 780 ± 0.1^a,b,c,e^ | | 881.9 ± 10.6^a,b,c,d^* | |
| ***Other micronutrients*** |  | |  | |  | |  | |  | |
| Vitamin A (µg RE/d) | 298 ± 6^c,d,e^ | | 320.1 ± 7.3^d,e^ | | 311.8 ± 8.1^a,d,e^ | | 364.3 ± 6.9^a,b,c,e^ | | 448.6 ± 9.1^a,b,c,d^* | |
| Vitamin D (mg/d) | 2.7 ± 0.1^c,d,e^ | | 2.7 ± 0.1^c,d,e^ | | 2.8 ± 0.2^a,b^ | | 5.1 ± 0.04^a,b^ | | 3 ± 0.1^a,b^* | |
| Zinc (mg/d) | 4.3 ± 0.1^b,c,d,e^ | | 4.9 ± 0.1^a,c,d,e^ | | 5.8 ± 0.2^a,b,d,e^ | | 6 ± 0.1^a,b,c,e^ | | 7.7 ± 0.2^a,b,c,d^* | |
| Iron (mg/d) | 5.8 ± 0.1^b,c,d,e^ | | 7.1 ± 0.1^a,c,d,e^ | | 8.1 ± 0.1^a,b,d,e^ | | 8.8 ± 0.1^a,b,c,e^ | | 10.4 ± 0.2^a,b,c,d^* | |
| Sodium (mg/d) | 673.7 ± 14.7^b,c,d,e^ | | 7910.7 ± 15^a,c,d,e^ | | 918.2 ± 17.6^a,b,d,e^ | | 997.6 ± 20^a,b,c,e^ | | 1287.8 ± 25^a,b,c,d^* | |
| Selenium (µg/d) | 64.7 ± 0.8^b,c,d,e^ | | 79.2 ± 1.1^a,c,d,e^ | | 86.2 ± 1.3^a,b,d,e^ | | 93.4 ± 1.3^a,b,c,e^ | | 106.9 ± 1.2^a,b,c,d^* | |
| Magnesium (mg/d) | 133.3 ± 1.6^b,c,d,e^ | | 140.1 ± 1.8^a,e^ | | 146.4 ± 2.3^a,e^ | | 144.9 ± 2.3^a,e^ | | 173.7 ± 5.1^a,b,c,d^* | |
| Potassium (mg/d) | 845.2 ± 11.1^b,c,d,e^ | | 895.8 ± 10.8^a,c,d,e^ | | 932.2 ± 12.6^a,b,d,e^ | | 998.6 ± 15.1^a,b,c,e^ | | 1184.9 ± 15.1^a,b,c,d^* | |

Significantly different from ^a^ poorest, ^b^ poor, ^c^ middle, ^d^ rich and ^e^ richest, P < 0.05 by using hypothesis testing to compare two population proportion with Bonferroni Error Correction. ^NS^ not significantly different.

Supplementary Table 4. Mean usual energy and nutrient intakes among adolescents aged 13-18 year from the 2013 NNS by wealth quintile

|  | | | **Mean ± SE** | | | | | | | | | | | |
| --- | --- | --- | --- | --- | --- | --- | --- | --- | --- | --- | --- | --- | --- | --- |
| **Nutrients** | **Poorest (n=1333)** | | | | **Poor (n=1203)** | | | **Middle (n=1082)** | | | **Rich (n=903)** | | | **Richest (n=781)** |
| Energy intake (Kcal/d) | 1514 ± 14.7^b,c,d,e^ | | | 1667 ± 16.2^a,c,d,e^ | | | 1807 ± 17.4^a,b,d,e^ | | | 1877 ± 19.6^a,b,c,e^ | | | 1952 ± 17.4^a,b,c,d^* | |
| **Macronutrients** |  | | |  | | |  | | |  | | |  | |
| Total fat (g/d) | 18.5 ± 0.3^b,c,d,e^ | | | 24.7 ± 0.4^a,c,d,e^ | | | 30.2 ± 0.5^a,b,d,e^ | | | 38.5 ± 0.5^a,b,c,e^ | | | 49.4 ± 0.5^a,b,c,d^* | |
| Saturated fat (g/d) | 8.5 ± 0.2^a,b,c,d^ | | | 11 ± 0.2^a,c,d,e^ | | | 13.6 ± 0.2^a,b,d,e^ | | | 16.8 ± 0.1^a,b,c,e^ | | | 21.6 ± 0.2^a,b,c,d^* | |
| Protein (g/d) | 48 ± 0.5^b,c,d,e^ | | | 54 ± 0.5^a,c,d,e^ | | | 58.9 ± 0.5^a,b,d,e^ | | | 62.5 ± 0.6^a,b,c,e^ | | | 68.7 ± 0.6^a,b,c,d^* | |
| Carbohydrate (g/d) | 291 ± 3^b,c,d,e^ | | | 310 ± 3.1^a,c,d^ | | | 327 ± 3.3^a,b,e^ | | | 324 ± 4.2^a,b^ | | | 312 ± 3.5^a,c^* | |
| Total sugars (g/d) | 20 ± 0.3^b,c,d,e^ | | | 21.8 ± 0.3^a,c,d,e^ | | | 25.4 ± 0.4^a,b,d,e^ | | | 29.8 ± 0.6^a,b,c,e^ | | | 35.9 ± 0.7^a,b,c,d^* | |
| Dietary fiber (g/d) | 9.2 ± 0.1^b,d,e^ | | | 8.6 ± 0.1^a,e^ | | | 8.9 ± 0.1^e^ | | | 8.6 ± 0.1^a,e^ | | | 8.1 ± 0.1^a,b,c,d^* | |
| **As percentage of total energy** | |  | | | |  | | |  | | |  | | |
| Total Fat (%) | 10.6 ± 0.1^b,c,d,e^ | | | 12.9 ± 0.1^a,c,d,e^ | | | 14.7 ± 0.2^a,b,d,e^ | | | 18.5 ± 0.2^a,b,c,e^ | | | 22.3 ± 0.2^a,b,c,d^* | |
| Protein (%) | 12.2 ± 0.1^b,c,d,e^ | | | 12.3 ± 0.1^a,c,d,e^ | | | 12.5 ± 0.1^a,b,d,e^ | | | 12.8 ± 0.1^a,b,c,e^ | | | 13.4 ± 0.1^a,b,c,d^* | |
| Carbohydrate (%) | 77.3 ± 0.2^b,c,d,e^ | | | 74.6 ± 0.2^a,c,d,e^ | | | 72.8 ± 0.2^a,b,d,e^ | | | 68.8 ± 0.2^a,b,c,e^ | | | 64.3 ± 0.2^a,b,c,d^* | |
| **Antioxidants** |  | | |  | | |  | | |  | | |  | |
| Vitamin C (mg/d) | 26.9 ± 0.5^b,c,d,e^ | | | 20.4 ± 0.4^a,c,d,e^ | | | 23.2 ± 0.4^a,b,d,e^ | | | 24.8 ± 0.5^a,b,c,e^ | | | 26.8 ± 0.4^a,b,c,d^* | |
| Vitamin E (mg/d) | 2.8 ± 0.1^c,d,e^ | | | 2.8 ± 0.04^c,d,e^ | | | 2.9 ± 0.04^a,b,e^ | | | 2.9 ± 0.04^a,b,e^ | | | 3.4 ± 0.04^a,b,c,d^* | |
| **B vitamins** |  | | |  | | |  | | |  | | |  | |
| Thiamine (mg/d) | 0.7 ± 0.01^b,c,d,e^ | | | 0.7 ± 0.01^a,c,d,e^ | | | 0.8 ± 0.01^a,b,d,e^ | | | 0.9 ± 0.01^a,b,c,e^ | | | 1 ± 0.01^a,b,c,d^* | |
| Riboflavin (mg/d) | 0.5 ± 0.01^b,c,d,e^ | | | 0.6 ± 0.01^a,c,d,e^ | | | 0.7 ± 0.01^a,b,d,e^ | | | 0.8 ± 0.01^a,b,c,e^ | | | 0.9 ± 0.01^a,b,c,d^* | |
| Niacin (mg/d) | 15.6 ± 0.2^b,c,d,e^ | | | 17.5 ± 0.2^a,c,d,e^ | | | 18.8 ± 0.2^a,b,d,e^ | | | 20 ± 0.2^a,b,c,e^ | | | 21 ± 0.2^a,b,c,d^* | |
| Vitamin B6 (mg/d) | 1.4 ± 0.02^c,d,e^ | | | 1.4 ± 0.02^c,d,e^ | | | 1.6 ± 0.02^a,b,d,e^ | | | 1.6 ± 0.03^a,b,c,e^ | | | 1.9 ± 0.03^a,b,c,d^* | |
| Vitamin B12 (µg/d) | 3.6 ± 0.04^b,e^ | | | 3.4 ± 0.1^a,d,e^ | | | 3.4 ± 0.04^d,e^ | | | 3.6 ± 0.1^b,c,e^ | | | 3.8 ± 0.04^a,b,c,d^* | |
| Folate (µg DFE) | 189 ± 3.3^b^ | | | 173 ± 2.6^a,e^ | | | 179 ± 2.5^NS^ | | | 176 ± 1.9^NS^ | | | 180 ± 2.8^b^* | |
| **Bone-related nutrients** |  | | |  | | |  | | |  | | |  | |
| Calcium (mg/d) | 271 ± 2.7^c,d,e^ | | | 268 ± 2.5^c,d,e^ | | | 297 ± 2.8^a,b,e^ | | | 294 ± 3.4^a,b,e^ | | | 331 ± 3.4^a,b,c,d^* | |
| Phosphorus (mg/d) | 711 ± 6.9^b,c,d,e^ | | | 777 ± 7.4^a,c,d,e^ | | | 846 ± 7.9^a,b,e^ | | | 860 ± 8.9^a,b,e^ | | | 912 ± 7.9^a,b,c,d^* | |
| Magnesium (mg) | 159 ± 1.6^c,d,e^ | | | 162 ± 1.5^c,d,e^ | | | 169 ± 1.7^a,b,e^ | | | 172 ± 2^a,b,e^ | | | 178 ± 1.8^a,b,c,d^* | |
| Vitamin D (µg/d) | 3.2 ± 0.1^d,e^ | | | 2.9 ± 0.04^d,e^ | | | 2.9 ± 0.04^d,e^ | | | 3 ± 0.04^a,b,c,e^ | | | 3 ± 0.04^a,b,c,d^* | |
| **Other micronutrients** |  | | |  | | |  | | |  | | |  | |
| Vitamin A (µg RE/d) | 342 ± 5.5^c,d,e^ | | | 340 ± 4.3^c,d,e^ | | | 364 ± 4.6^a,b,e^ | | | 378 ± 6.4^a,b,e^ | | | 396 ± 5.4^a,b,c,d^* | |
| Iron (mg/d) | 6.8 ± 0.1^b,c,d,e^ | | | 7.5 ± 0.1^a,c,d,e^ | | | 8.6 ± 0.1^a,b,d,e^ | | | 9.6 ± 0.1^a,b,c,e^ | | | 10.4 ± 0.1^a,b,c,d^* | |
| Zinc (mg/d) | 5 ± 0.1^b,c,d,e^ | | | 6.2 ± 0.1^a,d,e^ | | | 6.2 ± 0.1^a,d,e^ | | | 7.3 ± 0.1^a,b,c,e^ | | | 7.8 ± 0.1^a,b,c,d^* | |
| Sodium (mg/d) | 674 ± 9.6^b,c,d,e^ | | | 774 ± 11^a,c,d,e^ | | | 911 ± 11^a,b,d,e^ | | | 1003 ± 13^a,b,c,e^ | | | 1174 ± 14^a,b,c,d^* | |
| Selenium (µg/d) | 86 ± 0.9^b,c,d,e^ | | | 98 ± 1^a,c,d,e^ | | | 108 ± 1.1^a,b,d,e^ | | | 118 ± 1.3^a,b,c,e^ | | | 122 ± 1.1^a,b,c,d^* | |
| Potassium (mg/d) | 994 ± 9.4^b,c,d,e^ | | | 1035 ± 10^a,c,d,e^ | | | 1082 ± 10^a,b,d,e^ | | | 1144 ± 12^a,b,c,e^ | | | 1249 ± 11^a,b,c,d^* | |

Significantly different from ^a^ poorest, ^b^ poor, ^c^ middle, ^d^ rich and ^e^ richest, P < 0.05 by using hypothesis testing to compare two population proportion with Bonferroni Error Correction. ^NS^ not significantly different.
